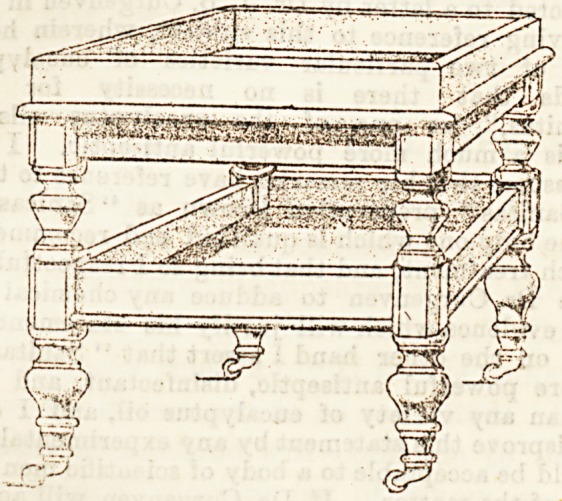# Ward Waggons

**Published:** 1893-07-15

**Authors:** 


### PRACTICAL DEPARTMENTS.

### WARD WAGGONS.

During a recent visit to the Temperance Hospital, Hampstead Road, we were much struck by the dinner waggons in use in the wards, and found from inquiries that they had been introduced by the Matron (who was most kind in supplying information), and had been made, we believe, from her design. By the kindness of Messrs. Levison, of New Oxford Street, we are enabled to give a sketch of a waggon similar in many respects to those which excited our admiration at the Temperance Hospital. As will be seen, the chief feature is that they are mounted on large castors, and are thus most convenient for taking patients' food straight from the lift into the ward, and where the number of patients to be provided for is not very large, the carving can easily be done upon them, avoiding any chance of spilling or upsetting the food, and keeping all the dinner requisites conveniently together. Both trays are tiled, and the tables are light in make, and move easily. Made in varnished pine, and fitted with white tiles or marble slab (preferably tiles, as they are lighter), they make quite a feature in a hospital ward, and will be found a great improvement on the tables of old days.

## Figures and Tables

**Figure f1:**